# Creation and validation of the Postpartum Specific Anxiety Scale Research Short-Form (PSAS-RSF)

**DOI:** 10.1007/s00737-021-01114-7

**Published:** 2021-04-26

**Authors:** Siân M. Davies, Paul Christiansen, Joanne A. Harrold, Sergio A. Silverio, Victoria Fallon

**Affiliations:** 1 School of Psychology, Liverpool John Moores University, Byrom Street, Liverpool, L3 3AF Merseyside UK; 2 Department of Psychology, University of Liverpool, Bedford Street South, Liverpool, L69 7ZA Merseyside UK; 3 Department of Women & Children’s Health, King’s College London, Westminster Bridge Road, London, SE1 7EH UK

**Keywords:** Psychometrics, Short-form, Postpartum anxiety, Perinatal mental health

## Abstract

The Postpartum Specific Anxiety Scale (PSAS) is a valid, reliable measure of postpartum anxiety (PPA). However, it contains 51 items, so is limited by its length. This study aimed to reduce the number of items in the PSAS, produce a small number of high-performing short-form tools, and confirm the factor structure of the most statistically and theoretically meaningful model. A pooled sample of English-speaking mothers (*N* = 2033) with infants up to 12 months were randomly split into three samples. (1) A principal component analysis (PCA) was conducted to initially reduce the items (*n* = 672). (2) Four short-form versions of varying length (informed by statistical, theoretical, lay-person, and expert-guided feedback) were developed and their factor structure examined (*n* = 673). (3) A final confirmatory factor analysis (CFA) was performed to confirm the factor structure of the PSAS Research Short-Form (PSAS-RSF) (*n* = 688). PCA and theoretical review reduced the items from 51 to 34 (version 1). Statistical review retained 22 items (version 2). Quantitative expert panel data retained 17 items (version 3). Qualitative expert panel data retained 16 items (version 4). The 16-item version was deemed the most theoretically and psychometrically robust. The resulting 16-item PSAS-RSF demonstrated good psychometric properties and reliability. The PSAS-RSF is the first brief research tool which has been validated to measure PPA. Our findings demonstrate it is theoretically meaningful, statistically robust, reliable, and valid. This study extends the use of the measure up to 12 months postpartum, offering broader opportunity for measurement while further enhancing accessibility through brevity.

## Introduction

Postpartum anxiety (PPA) has been associated with persistent and far-reaching outcomes for mothers and infants. These include associations with impaired maternal bond (Fallon et al. 2021), reduced maternal self-efficacy (Matthies et al. 2017), adverse infant feeding outcomes (Fallon et al. 2018), difficult infant temperament (Britton 2011), and poor infant developmental outcomes (Glasheen et al. 2010). PPA also poses an economic burden to the health care system and wider society (Bauer et al. 2014), with prevalence rates ranging from 13 to 40% in high income contexts (Field 2017). It is therefore crucial that symptoms of anxiety are correctly identified and appropriately measured.

PPA has been predominately identified and measured using scales such as the State-Trait Anxiety Inventory (STAI; Spielberger et al. 1983), and the Generalised Anxiety Disorder-7 (GAD-7; Spitzer et al. 2006). These were designed for use in general adult populations which is problematic in a childbearing context (National Collaborating Centre for Mental Health 2014). Items in the STAI such as *‘I feel rested’ *may inappropriately inflate anxiety scores as disrupted sleep is a normal aspect of motherhood (Galland et al. 2012). Conversely, general measures fail to capture specific maternal- and infant-focused concerns; consequently, low scores may not reflect the absence of symptoms (Phillips et al. 2009).

To overcome these psychometric issues, the 51-item Postpartum Specific Anxiety Scale (PSAS; Fallon et al. 2016) was developed and validated. It measures four domains of anxiety specific to the postpartum period: maternal competence and attachment anxieties; infant safety and welfare anxieties; practical infant care anxieties; and psychosocial adjustment to motherhood. To date, initial validity and reliability has been demonstrated in one large international English-speaking sample (Fallon et al. 2016), and more recently two Turkish samples (Duran 2020; Bayri Bingol et al. 2021). Predictive validity has also been confirmed in relation to infant feeding outcomes and behaviours (Fallon et al. 2018) and maternal bonding (Fallon et al. 2021), with the PSAS predicting unique variance in these outcomes after controlling for the STAI. At the time of writing, the PSAS has been requested for use, translation, and/or validation by 53 different research teams across 29 countries demonstrating a broad global interest in the tool and its ability to measure maternal- and infant-focused anxieties (see also Silverio et al. 2021). While the PSAS demonstrates good capability as a valid and reliable measure of PPA, there have been increasing requests for a shorter version to aid accessibility. There is potential to refine and reduce the measure further to perform as a short-form research tool which accurately identifies symptoms of PPA.

Building on the recently developed PSAS, this study aimed to:

i. Reduce the number of items in the PSAS using a principal component analysis (PCA)
ii. Produce a small number of high-performing short-form versions by weighting the contributions of different information sources (PSAS Working Group, statisticians, expert panel, psychometric properties)
iii. Confirm the factor structure of the most statistically and theoretically meaningful model.

## Method

### Participants

#### Mothers

A pooled dataset of mothers (*N* = 2033) with infants aged between birth and 12 months were compiled from five on-line surveys, which all used the PSAS. All mothers in the dataset were recruited via social media platforms with an advertisement containing a link to a Qualtrics surveys platform. Demographic information for the pooled sample can be found in Table 1.

**Table 1:** Maternal and infant characteristics (*N* = 2033)

Maternal characteristic	Value	Infant characteristic	Value
Maternal age (mean years ± SD)	32.25 (22.94)	Infant age (mean weeks ± SD)	13.02 (15.69)
Ethnicity (*N*/%)		Birth order (*N*/%)	
White	1935 (95.2)	1st	1221 (60.1)
Pakistani	4 (0.2)	2nd	644 (31.7)
Black Caribbean	3 (0.1)	3rd	128 (6.3)
Bangladeshi	5 (0.2)	4th	128 (6.3)
Black African	2 (0.1)	Timing of birth (*N*/%)	
Chinese	6 (0.3)	Premature (<37 weeks)	137 (6.5)
Indian	14 (0.7)	Early term (>37 < 39 weeks)	396 (19.4)
Black other	1 (0.0)	Full term (39 weeks)	522 (25.7)
Other	56 (2.8)	Post term (>40 weeks)	978 (48.1)
Prefer not to say	7 (0.3)	Multiple birth (*N*/%)	
Marital status (*N*/%)		Yes	23 (1.1)
Married	1248 (61.4)	No	2010 (98.9)
Co-habiting	741 (36.4)	Current feeding method (*N*/%)	
Divorced	2 (0.1)	Exclusively breastfeeding (100%)	1056 (51.9)
Widowed	0 (0.0)	Predominantly breastmilk (over 80%) with a little formula milk (20%)	175 (8.6)
Separated	4 (0.2)	Mainly breastmilk (50–80%) with some formula milk	36 (1.8)
Single	38 (1.9)	A combination of both breastmilk (50%) and formula milk (50%)	36 (1.8)
Occupation (*N*/%)		Mainly formula milk (50–80%) with some breastmilk	21 (1.0)
Managers, directors, senior officials	247 (12.1)	Predominantly formula milk (over 80%) with a little breastmilk (20%)	34 (1.7)
Professionals	874 (43.0)	Exclusively formula feeding (100%)	675 (33.2)
Associate professionals and technical	59 (2.9)		
Administrative and secretarial	297 (14.6)		
Skilled trade	43 (2.1)		
Caring, leisure, and other service	337 (16.6)		
Process, plant, and machine operatives	4 (0.2)		
Elementary	27 (1.3)		
Not in paid occupation	145 (7.1)		
Education attainment (*N*/%)			
Postgraduate education	500 (24.6)		
Undergraduate education	899 (44.2)		
A-levels or college equivalent	390 (19.2)		
GCSEs or secondary school equivalent	168 (8.3)		
No qualifications	24 (1.2)		
Other qualification	52 (2.6)		
Current diagnosis of anxiety (*N*/%)			
Yes	378 (18.6)		
No	1642 (80.2)		
Prefer not to say	13 (0.6)		
Current diagnosis of depression (*N*/%)			
Yes	290 (14.3)		
No	1734 (85.3)		
Prefer not to say	9 (0.4)		

#### Expert panel

A panel consisting of 16 individuals (psychologists, perinatal researchers, midwives, health visitors, statisticians, psychometricians, and parents) were recruited via purposive sampling.

### Design and procedure

#### Mothers

**Fig. 1:**
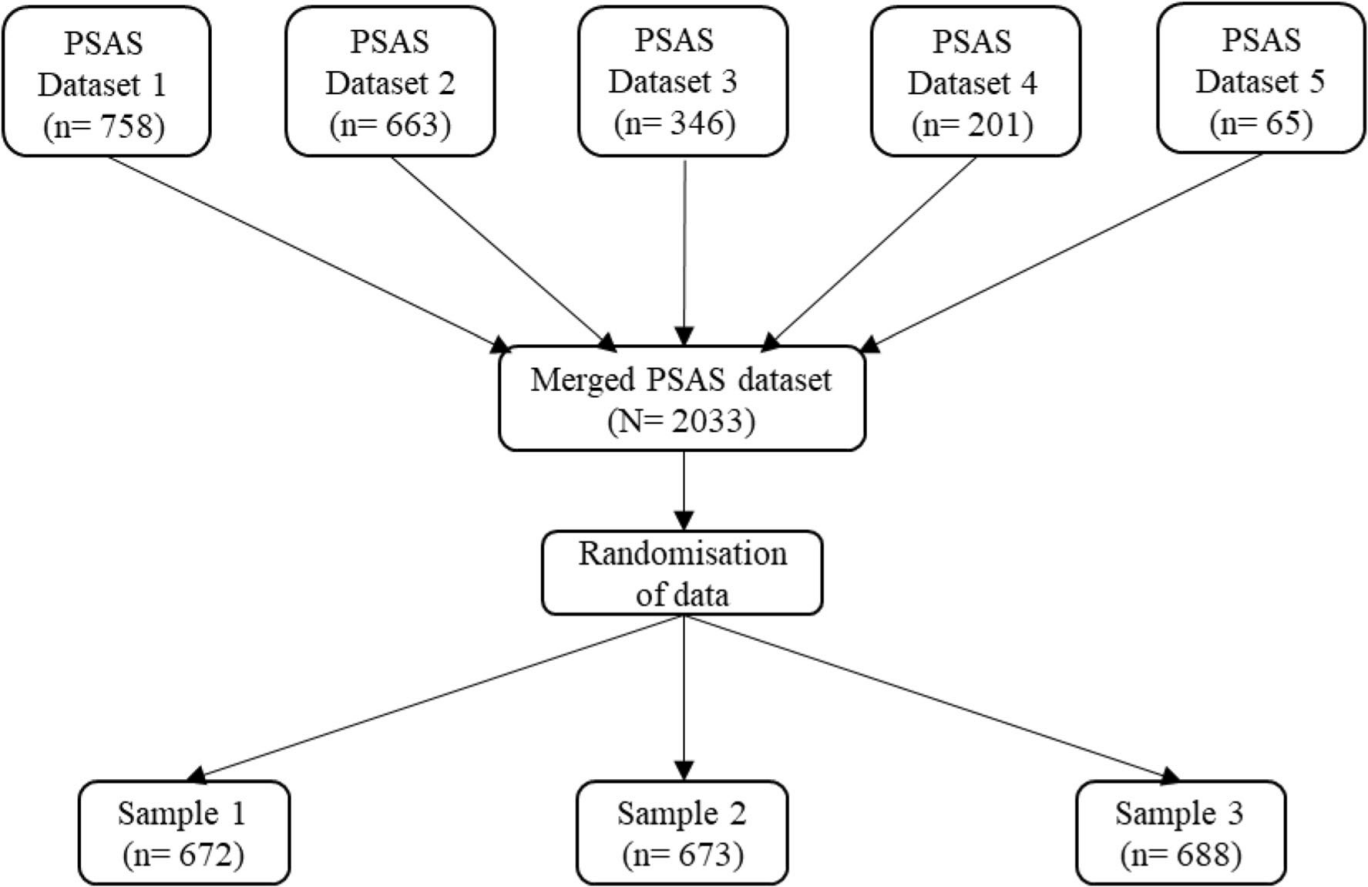
Flow chart to demonstrate how data was prepared for analyses

Five cross-sectional, on-line surveys were combined with data collected between 2017 and 2020 (see Fig. 1). Prior to each of the surveys, participants who met the eligibility criteria gave informed consent. Upon completion of the surveys, participants were provided with a full electronic debrief with signposting to appropriate support information and redirected to a £25 prize draw.

#### Expert panel

A cross-sectional on-line survey was distributed via e-mail with a Qualtrics link after development of the first preliminary version (see the method of analysis section). Prior to the survey, expert panel members gave informed consent.

### Measures

#### Demographic questions

Maternal-related demographic questions were asked at the beginning of each survey including maternal age, ethnicity, marital status, occupation, educational attainment, clinical diagnosis of anxiety, and clinical diagnosis of depression. Infant-related demographic questions were additionally asked including infant age, multiple birth status, birth order, gestational age at delivery, and feeding practices.

#### Postpartum Specific Anxiety Scale (PSAS; Fallon et al. 2016)

The PSAS is a 51-item scale that examines the frequency of maternal- and infant-focused anxieties experienced by women over the last week during the first year following birth. It assesses four components of anxiety, specific to the postpartum period. Factor 1 (maternal competence and attachment anxieties) contains 15 items addressing anxieties relating to maternal self-efficacy, parenting competence, and the mother-infant relationship. Factor 2 (infant safety and welfare anxieties) contains 11 items which relate to fears about infant illnesses, accidents, and cot death. Factor 3 (practical infant care anxieties) contains 7 items which address anxieties which relate to infant care such as feeding, sleeping, and general routine. Finally, factor 4 (psychosocial adjustment to motherhood) contains 18 items addressing adjustment concerns following birth of the infant regarding management of personal appearance, relationships and support, work, finances, and sleep. Each item is scored between 1 and 4 with a maximum score of 204. The PSAS demonstrates excellent reliability in the current study (McDonald’s *ω* = .96).

### Expert panel survey items

Each panel member (blinded from other members’ feedback) rated the relevance and comprehensibility of each item using a Likert scale (0–5; 0 = not at all relevant or comprehensive and 5 = highly relevant or comprehensive). Open questions asked the panel to feedback on items individually and suggest removal or retention of items. They were each asked how many items they believed a short-form research tool should contain. Finally, they rated the measure overall in terms of how easy it was to understand and to complete (0–10; 0 = not at all easy to understand and complete and 10 = extremely easy to understand or complete).

### Ethics

Ethical approvals were sought and granted by the Institute of Psychology, Health and Society Research Ethics Committee at the University of Liverpool (refs: IPHS/2014; IPHS/5328; IPHS/3647; IPHS/1415/LB/233).

### Method of analysis

The data was split into three samples using randomisation in SPSS (see Fig. 1). An initial PCA (*n* = 672) was performed to reduce the number of items in the PSAS by identifying low-performing or cross-loading items. The PSAS Working Group initially reviewed and reduced the items to produce version 1. A statistician then reviewed and reduced version 1 to produce version 2. An expert panel then reviewed version 1 to provide quantitative feedback on face and content validity in order to produce version 3. Qualitative feedback from the expert panel produced version 4. Confirmatory factor analyses (CFA) for all four preliminary versions (*n* = 673) were then conducted to identify the most statistically and theoretically meaningful version. The factor structure of the final PSAS-RSF was then confirmed in the final sample (*n* = 688).

#### Principal component analysis (*n* = 672)

A parallel analysis was performed to obtain the number of factors present in the data. Then, a PCA using oblique (oblimin) rotation (set to the number of factors found by the parallel analysis) was conducted on the polychoric correlation matrix due to the data being ordinal.

#### Confirmatory factor analyses (sample 2 *n* = 673; sample 3 *n* = 688)

Confirmatory factor analyses were performed on each of the four proposed versions using R version 4.0 using diagonal weighted least squares estimation as the data was ordinal (see Mîndrilã 2010). Items were free to load onto their corresponding latent factors, and latent factors were free to correlate with one another. Model fit was assessed using the comparative fit index (CFI) and the Tucker-Lewis Index (TLI), with values of above .90 being deemed acceptable and values of .95 deemed good (Hu and Bentler 1999). For the root mean square error of approximation (RMSEA), values of .05 and under are deemed good, values of .08 and under are deemed fair, values between .08 and .10 are deemed mediocre, and values over .10 are considered a poor fit (MacCallum et al. 1996). For the standardised root mean square residual (SRMR), values less than .08 are considered a good fit (Hu and Bentler 1999). Modification indices were inspected; covariance pathways were added between error terms (if in excess of 20, providing they were conceptually appropriate, and the items loaded onto the same factor). A final CFA was then performed on the third random sample to confirm the final factor structure of the PSAS-RSF.

#### Internal consistency

Internal reliability of the full scale and each subscale was estimated with McDonald’s *ω* (see Revelle and Zinbarg 2009).

## Results

### Factor structure of the PSAS

The factor structure of the PSAS (Table 2) was assessed using all the participants in sample 1 (*n* = 672). First, the parallel analysis suggested there were four factors which is consistent with the original 51-item measure (Fallon et al. 2016) and the 12-item crisis short-form (Silverio et al. 2021). The PCA demonstrated that the sampling adequacy for the scale was excellent (KMO = 0.95) and Bartlett’s test of sphericity indicated sufficient correlations for PCA (*χ*^2^(1275) = 15,634.93, *p* < .001). The PCA revealed a four-factor solution explaining a combined total of 44% of the variance in the data. The four factors had good to excellent reliability, with McDonald’s *ω* ranging from .78 to .90. Furthermore, the overall scale had excellent reliability (McDonald’s *ω* = .96). An exploratory factor analysis (EFA) was also conducted and produced analogous results across all items.

**Table 2:** Factor structure of the PSAS

	Rotated components			
Scale Item	1	2	3	4
Factor 1: Psychosocial adjustment to motherhood anxieties				
I have worried more about completing household chores than before my baby was born	**0.70**	−0.02	−0.14	0.14
I have felt resentment towards my partner	**0.68**	−0.13	0.04	−0.03
I have been less able to concentrate on simple tasks than before my baby was born	**0.66**	0.10	−0.08	0.08
I have felt that I have had less control over my day than before my baby was born	**0.63**	0.18	−0.10	−0.12
I have felt unable to juggle motherhood with other responsibilities	**0.61**	0.06	0.01	−0.09
I have worried that my partner finds me less attractive than before my baby was born	**0.61**	0.00	−0.13	0.22
I have felt isolated from family and friends	**0.60**	0.01	0.09	0.13
I have worried more about my relationship with my partner than before my baby was born	**0.60**	−0.11	0.17	0.10
I have felt that I am not the parent I want to be	**0.59**	0.17	0.20	−0.13
I have felt tired even after a good amount of rest	**0.58**	0.00	−0.07	0.18
I have felt that I do not get enough support	**0.56**	−0.06	0.16	0.04
I have worried more about my relationship with my family than before my baby was born	**0.53**	−0.15	0.30	0.09
I have worried that I am not going to get enough sleep	**0.51**	0.06	0.04	−0.08
I have felt that other mothers are coping with their babies better than me	**0.51**	0.42	0.14	−0.14
I have felt that motherhood is much harder than expected	**0.50**	0.14	0.18	−0.15
I have worried more about my finances than before my baby was born	**0.46**	0.02	−0.13	0.30
I have worried more about my appearance than before my baby was born	**0.44**	0.03	−0.18	0.15
I have had difficulty sleeping even when I have had the chance to	**0.44**	0.06	0.07	0.29
I have felt that I should not need help to look after my baby	**0.41**	0.03	0.09	0.07
I have worried that other people think my parenting skills are inadequate	**0.41**	0.27	0.22	0.01
I have worried more about my relationship with my friends than before my baby was born	**0.38**	−0.11	0.29	0.25
Factor 2: Practical infant care anxieties				
I have worried about my baby’s milk intake	−0.05	**0.71**	−0.08	0.16
I have worried about the way that I feed my baby	−0.04	**0.66**	0.03	0.06
I have worried about my baby’s weight	−0.25	**0.65**	−0.04	0.25
I have worried about the length of time that my baby sleeps	0.30	**0.54**	−0.12	−0.22
I have worried that my baby is less content than other babies	0.17	**0.53**	0.15	−0.09
I have worried about my baby’s health even after reassurance from others	0.06	**0.52**	0.06	0.39
I have used the internet for reassurance about my baby’s health	0.12	**0.48**	−0.17	0.25
I have worried that my baby is not developing as quickly as other babies	0.04	**0.47**	0.10	0.21
I have felt unconfident or incapable of meeting my baby’s basic care needs	0.08	**0.46**	0.38	−0.09
I have worried about being unable to settle my baby	0.31	**0.45**	0.20	−0.20
I have worried that my baby feels more content in someone else’s care	0.13	**0.37**	0.24	−0.08
I have worried about how I will cope with my baby when others are not around to support me	0.30	**0.35**	0.19	−0.14
I have worried about the bond I have with my baby	0.31	**0.35**	0.28	−0.11
I have worried about getting my baby into a routine	0.16	**0.34**	0.27	0.04
I have worried about accidentally harming my baby	0.25	**0.28**	0.13	0.16
Factor 3: Maternal competence and attachment anxieties				
I have felt that my baby would be better cared for my someone else	−0.09	−0.02	**0.88**	0.02
I have had negative thoughts about my relationship with my baby	0.03	0.00	**0.82**	−0.02
I have worried I will not know what to do when my baby cries	−0.02	0.14	**0.67**	−0.03
I have worried that I will become too ill to care for my baby	0.07	−0.04	**0.56**	0.31
I have felt that when I do get help it is not beneficial	0.28	−0.03	**0.46**	0.26
I have worried that my baby is picking up on my anxieties	0.34	0.12	**0.44**	0.14
Factor 4: Infant safety and welfare anxieties				
I have repeatedly checked on my sleeping baby	0.08	0.13	−0.12	**0.64**
I have worried that my baby will stop breathing while sleeping	0.03	0.14	0.11	**0.64**
I have felt frightened when my baby is not with me	0.16	0.04	0.14	**0.61**
I have thought of ways to avoid exposing my baby to germs	−0.09	0.13	0.16	**0.53**
I have worried about my baby being accidentally harmed by someone or something else	0.09	−0.02	0.38	**0.50**
I have worried about leaving my baby in a childcare setting	0.20	0.16	−0.09	**0.45**
I have felt a greater need to do things in a certain way or order than before my baby was born	0.23	0.03	0.19	**0.39**
I have worried about returning to work	0.32	0.00	−0.14	**0.38**
I have not taken part in an everyday activity with my baby because I fear they may come to harm	0.17	0.21	0.24	**0.24**
% of variance explained	17	10	9	8
McDonald’s *ω*	.90	.85	.78	.88

All significant loadings in bold

#### Version 1 production: PSAS Working Group review

The UK-based PSAS Working Group conducted an initial theoretical review of the factor loadings with consideration to the psychometric work conducted to date on the original PSAS. This suggested the factor structure of the PSAS-RSF replicated that of the original 51-item PSAS. Thirty-four items loaded onto the same factors as the original 51-item PSAS at >.30. Fifteen items cross-loaded onto at least one other factor at >.30 and two items were low-performing at <.30. A comparison of psychometric properties highlighted that these items also had sub-optimal factor loadings or cross-loaded in the original 51-item PSAS. These items were removed resulting in the 34-item scale (version 1).

#### Version 2 production: statistical review

A statistical review of version 1 was then performed by PC (PSAS statistician). Factor loadings and items at ≥.40 were retained to create a 22-item alternative version.

#### Expert panel

Descriptive analyses were conducted on the quantitative expert panel data. The measure was deemed relevant in terms of the overall construct (*M* = 121.00, ±11.55; maximum score = 170) and the individual factors (*M* = 121.19, ±10.11; maximum score = 170). It was considered easy to understand (*M* = 8.53, ±2.26; maximum score = 10) and complete (*M* = 8.13, ±2.99; maximum score = 10). The panel believed that an ideal short form should contain ~ 20 items. Quantitative and qualitative expert panel data were used to inform versions 3 and 4.

#### Version 3 production: face and content validity review

The quantitative data from the expert panel was reviewed for applicability and relevance to construct and factor on the 34-item scale (version 1). Six items that were not universally applicable to all women such as *‘I have felt resentment towards my partner’* were discarded. Eleven items were removed on the basis they were not considered highly relevant to both PPA and relevant to the factor to which they belonged, creating a 17-item scale (version 3).

#### Version 4 production: qualitative review

Qualitative feedback from the panel on version 1 was then reviewed for each item. Positive comments informed the retention of items and negative comments informed the removal of items, creating a 16-item scale (version 4). For example, item 15 *‘I have had difficulty sleeping even when I have had the chance to’ *was removed altogether due to a theoretically similar item being preferred. Item 22 *‘I have worried that my baby is not developing as quickly as other babies’* was replaced with item 23 *‘I have worried about getting my baby into a routine’,* due to item 23 having a more positive qualitative appraisal by the expert panel.

### Confirmation of factor structure for versions 1, 2, 3, and 4 (*n* = 673)

Corresponding items for each version can be found in Table 3. Model fit data for each version is provided in Table 4.

**Table 3:** Items retained in each high-performing version

	Version			
Scale item	1	2	3	4
I have felt unable to juggle motherhood with my other responsibilities.	*	*	*	*
I have worried more about my relationship with my family than before my baby was born.	*			
I have worried about accidentally harming my baby.				
I have worried about how I will cope with my baby when others are not around to support me.				
I have felt that I do not get enough support.	*	*		
I have been less able to concentrate on simple tasks than before my baby was born.	*	*		
I have felt that I should not need help to look after my baby.				
I have felt frightened when my baby is not with me.	*	*	*	*
I have worried I will not know what to do when my baby cries.	*	*	*	*
I have worried more about my relationship with my partner than before my baby was born.	*	*		
I have worried that my baby feels more content in someone else’s care.				
I have felt isolated from my family and friends.	*	*		
I have worried about my baby’s weight.	*		*	*
I have worried about getting my baby into a routine.	*			*
I have worried that I will become too ill to care for my baby.				
I have worried about my baby being accidentally harmed by someone or something.	*		*	*
I have felt unconfident or incapable of meeting my baby’s basic care needs.				
I have worried about being unable to settle my baby.				
I have felt a greater need to do things in a certain way or order than before my baby was born.	*	*		
I have had negative thoughts about my relationship with my baby.	*	*	*	*
I have worried more about my relationship with my friends than before my baby was born.	*			
I have thought of ways to avoid exposing my baby to germs.	*	*		
I have worried that my baby is less content than other babies.				
I have felt that other mothers are coping with their babies better than me.				
I have felt that I am not the parent I want to be.				
I have worried more about completing household chores than before my baby was born.	*	*		
I have not taken part in an everyday activity with my baby because I fear they may come to harm.				
I have worried about my baby’s milk intake.	*	*	*	*
I have felt that I have had less control over my day than before my baby was born.	*	*	*	*
I have worried more about my finances than before my baby was born.	*		*	*
I have worried about my baby’s health even after reassurance from others.				
I have felt that when I do get help it is not beneficial.				
I have worried that my baby will stop breathing while sleeping.	*	*	*	*
I have used the internet for reassurance about my baby’s health.	*	*		
I have worried about leaving my baby in a childcare setting.	*			
I have felt that my baby would be better cared for by someone else.	*	*	*	*
I have worried that I am not going to get enough sleep.	*	*	*	*
I have felt that motherhood is much harder than I expected.				
I have worried that my baby is picking up on my anxieties.	*		*	*
I have worried about the bond that I have with my baby.				
I have worried about the length of time that my baby sleeps.	*			*
I have worried about returning to work.				
I have worried more about my appearance than before my baby was born.	*			
I have had difficulty sleeping even when I have had the chance to.	*		*	
I have worried that other people think that my parenting skills are inadequate.				
I have worried that my partner finds me less attractive than before my baby was born.	*	*		
I have worried that my baby is not developing as quickly as other babies.	*		*	
I have felt resentment towards my partner.	*	*		
I have worried about the way that I feed my baby.	*	*	*	
I have repeatedly checked on my sleeping baby.	*	*	*	*
I have felt tired even after a good amount of rest.	*	*

**Table 4:** Confirmation of factor structure for versions 1, 2, 3, and 4 (*n* = 673)

Goodness of fit indices	Version 1	Version 1**	Version 2	Version 2*	Version 3	Version 3*	Version 4	Version 4**
CFI	.95	.96	.96	.97	.96	.97	.93	.96
TLI	.94	.95	.95	.96	.95	.96	.91	.95
RMSEA	0.05	0.05	0.05	0.04	0.05	0.04	0.07	0.05
SRMR	0.07	0.07	0.06	0.06	0.07	0.06	0.08	0.07
McDonald’s ω	.92	–	.89	–	.87	–	.87	–

*CFI*, comparative fit index; *TLI*, Tucker-Lewis Index; *RMSEA*, root mean square of error; *SRMR*, standardised root mean residual

^**^Model fit improvement following the addition of covariances between two pairs of residuals

^*^Model fit improvement following the addition of covariances between one pair of residuals

### PSAS-RSF final version

Based on a combination of the initial PCA, expert panel results, and the CFA, the PSAS Working Group selected 16-item version 4 as the most statistically robust and theoretically meaningful measure.

### Confirmation of factor structure for PSAS-RSF (*n* = 688; see Table 5)

**Fig. 2:**
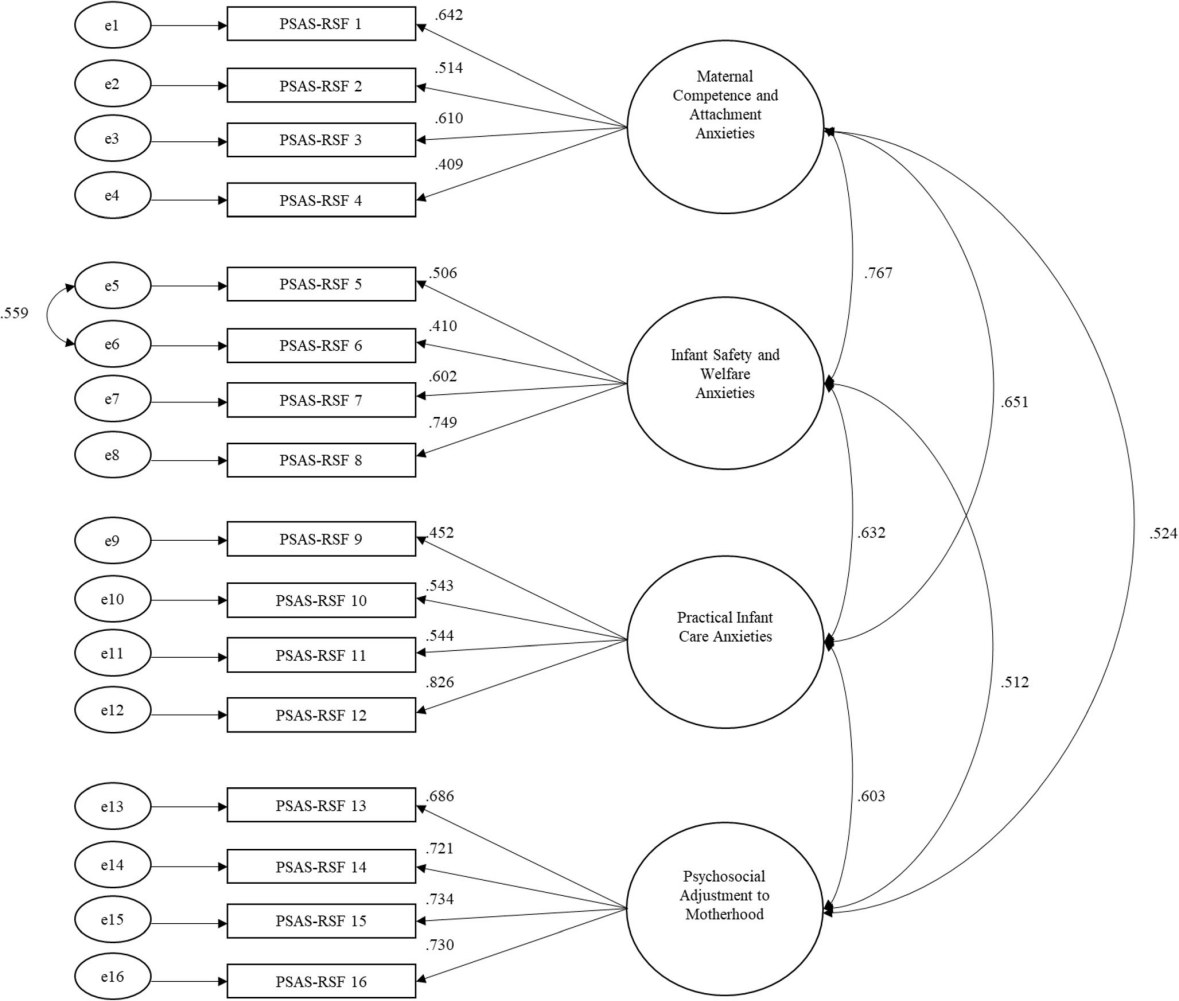
Standardised factor loading

The initial version was a good fit of the data (CFI = .96, TLI = .96, RMSEA = 0.05, SRMR = 0.07). Modification indices indicated that a covariance should be added between 1 pair of residuals. As a result, the model fit improved (CFI = .98, TLI = .97, RMSEA = 0.04, SRMR = 0.06). All items significantly loaded onto each factor (*p* < .001; see Fig. 2 for the standardised factor loadings). The four factors had moderate to good reliability, with McDonald’s *ω* ranging from .65 to .80. The overall scale demonstrated good reliability (McDonald’s *ω* = .88).

**Table 5:** Factor structure of the PSAS-RSF

	Rotated components			
Scale item	1	2	3	4
Factor 1: Psychosocial adjustment to motherhood				
1. I have felt that I have had less control over my day than before my baby was born	**0.63**	0.18	−0.10	−0.12
2. I have felt unable to juggle motherhood with other responsibilities	**0.61**	0.06	0.01	−0.09
3. I have worried that I am not going to get enough sleep	**0.51**	0.06	0.04	−0.08
4. I have worried more about my finances than before my baby was born	**0.46**	0.02	−0.13	0.30
Factor 2: practical infant care anxieties				
5. I have worried about my baby’s milk intake	−0.05	**0.71**	−0.08	0.16
6. I have worried about my baby’s weight	−0.25	**0.65**	−0.04	0.25
7. I have worried about the length of time by baby sleeps	0.30	**0.54**	−0.12	−0.22
8. I have worried about getting my baby into a routine	0.16	**0.34**	0.27	0.04
Factor 3: maternal competence and attachment anxieties				
9. I have felt that my baby would be better cared for my someone else	−0.09	−0.02	**0.88**	0.02
10. I have had negative thoughts about my relationship with my baby	0.03	0.00	**0.82**	−0.02
11. I have worried I will not know what to do when my baby cries	−0.02	0.14	**0.67**	−0.03
12. I have worried that my baby is picking up on my anxieties	0.34	0.12	**0.44**	0.14
Factor 4: infant safety and welfare anxieties				
13. I have repeatedly checked on my sleeping baby	0.08	0.13	−0.12	**0.64**
14. I have worried that my baby will stop breathing while sleeping	0.03	0.14	0.11	**0.64**
15. I have felt frightened when my baby is not with me	0.16	0.04	0.14	**0.61**
16. I have worried about my baby being accidentally harmed by someone or something else	0.09	−0.02	0.38	**0.50**
McDonald’s ω	0.60	0.80	0.82	0.85

All significant loadings in bold

## Discussion

The primary aim of the current study was to develop a shorter and more accessible measure of postpartum specific anxiety and to confirm the factor structure of this measure to aid research measurement in childbearing populations. Exploratory psychometric analyses (*n* = 672) enabled the initial reduction from 51 to 34 items. Then, utilising multiple sources of information, 22-item, 17-item, and 16-item versions were produced. The psychometric properties of all four versions were examined in a second sample of English-speaking postpartum mothers (*n* = 673), demonstrating good model fits. Furthermore, theoretical and statistical examination of these results suggested that a 16-item tool was the most robust. The factor structure of the final 16-item PSAS-RSF was then confirmed in a third sample of mothers (*n* = 688) and demonstrated an overall good fit of the data. The PSAS-RSF was found to be a theoretically meaningful, statistically robust short-form tool with initial supporting evidence for reliability and construct validity for measuring postpartum specific anxiety.

The PSAS-RSF is a multidimensional instrument, comprising of four distinct, but correlated domains of (1) psychosocial adjustment to motherhood; (2) practical infant care anxieties; (3) maternal competence and attachment anxieties; and (4) infant safety and welfare anxieties. Despite reducing the original PSAS by 35 items and the domains now consisting of comparatively fewer items (four items on each subscale), the measure demonstrated good construct validity and overall reliability (total and subscales). Furthermore, the overall factor structure and individual items retained on each factor mirrored that of the original PSAS. The items retained on factor one related to anxieties concerning managing the household since the birth of the baby including lack of control during the day (item 1), sleep management at night (item 3), finances (item 4), and general responsibilities (item 2). A recent study examining women with no symptoms of anxiety also found that environmental mastery including the ability to control an array of external activities was key to well-being, which provides interesting divergent support to this construct (Grussu et al. 2020). Those on factor two pertain to infant regulatory concerns including infant feeding (item 5), weight (item 6), sleep (item 7), and routine (item 8). Supporting evidence from clinical samples demonstrates that maternal anxiety is consistently associated with infant regulatory issues as both an antecedent and a consequence (Lonstein 2007; Richter and Reck 2013). Factor three retained items concerning maternal self-efficacy (items 9 and 11) and relational anxieties (items 10 and 12). Perceived self-efficacy is a fundamental construct in anxiety arousal (Bandura 1988). Current and previous anxiety in new mothers has been found to significantly negatively impact maternal self-confidence which offers convergent support (Reck et al. 2012). Furthermore, the predictive validity of the original PSAS has been confirmed in relation to infant feeding outcomes (regulatory in nature) and maternal bonding behaviours (relational in nature) (Fallon et al. 2018; Fallon et al. 2021, respectively). The items remaining in the final factor concerned accidental harm when separated from the infant (i.e. during sleep [items 13 and 14] or when in others’ care [items 15 and 16]). State anxiety has been associated with perceptions of infant vulnerability and maternal reactions include overprotection, separation anxiety, and excessive concerns about infant health (Kerruish et al. 2005) which all directly align with this construct. The current study (and previous PSAS work) has demonstrated that these constructs of PPA are stable across multiple samples and embedded in the research literature. As such, use of the PSAS-RSF offers opportunity for effective measurement of maternal- and infant-focused anxiety in the first postpartum year.

Seventeen of the surplus items that were not retained cross-loaded onto more than one factor in both this study and the original validation of the PSAS (Fallon et al. 2016). This suggests that the PSAS-RSF is psychometrically reliable as lower performing items behaved consistently across both validity studies. A further six optional items relating to work, partners, family, and friends were removed in order for the PSAS-RSF to be universally applicable to all women’s circumstances. An expert panel study informed the removal of the remaining twelve items on the basis of quantitative face and content validity and qualitative feedback. This multi-faceted approach using a variety of data sources allowed the reduction of items to be informed by statistical and theoretical knowledge, and both lay-person and expert-guided decision-making (Streiner et al. 2015).

A common consequence of creating short-form measures is reduced psychometric qualities in comparison to their original counterparts due to the fact that there are fewer items measuring the construct (Widaman et al. 2011). While the PSAS offers a more detailed assessment of anxiety, the PSAS-RSF performed comparably well to its parent form and reduces potential response burden, particularly when used alongside a battery of measures or in a health care setting (see also PSAS-RSF-C; Silverio et al. 2021). Furthermore, the original PSAS was validated in a sample of mothers of infants <6 months old. This study extends the valid use of the measure up to 12 months postpartum, offering broader opportunity for measurement while further enhancing accessibility through brevity.

### Strengths, limitations, and future directions

Commonly, psychometric studies of mood (including the original PSAS) use analyses appropriate for interval-level data which do not reflect the ordinal nature of subjective psychological states. The use of the polychoric correlation matrix is an ordinal level method of analysis that mitigates this issue (Kolenikov and Angeles 2004). Furthermore, the current study also has adopted the use of McDonald’s omega to assess reliability which is statistically more robust than Cronbach’s alpha (Revelle and Zinbarg 2009). The use of a large (>2000) pooled dataset enabled both exploratory and confirmatory psychometric analyses on separate randomised samples. While the sample was large, like other on-line data collection methods in perinatal populations (Vignato et al. 2019), it lacked diversity which resulted in participants being predominately white, married, university-level educated, professional women. Recommendations to validate the original PSAS in ethnically and socio-economically diverse populations have already been made (Fallon et al. 2016), but the accessible nature of the PSAS-RSF may make reaching these groups more viable.

There has been broad global interest in cultural adaptation, translation, and validation of the PSAS with two studies already published in Turkey (Duran 2020; Bayri Bingol et al. 2021). We envisage a similar level of interest in the PSAS-RSF, particularly given the brief nature of the 16-item tool. Factorial invariance has yet to be examined and future studies should assess whether the factorial structure is stable across different groups (e.g. cross-culturally, by parity, and social class). Predictive validity work using the PSAS demonstrates that it predicts unique variance (after accounting for general anxiety) in maternal and infant outcomes (Fallon et al. 2018; Fallon et al. 2021). These findings need replication using the PSAS-RSF.

## Conclusion

This study offers a theoretically meaningful, statistically robust short-form tool with initial supporting evidence for reliability and construct validity for measuring postpartum specific anxiety. To the best of our knowledge, the PSAS-RSF is the first brief research tool which has been validated to measure PPA during the first year following birth. The PSAS-RSF can be utilised as part of a battery of psychological measures or in a healthcare setting with minimal response burden to participants. The ability to quickly and accurately measure maternal- and infant-focused anxieties will aid further understanding about the course, nature, antecedents, and outcomes of anxiety in the first year after birth.

## Data Availability

The data used in this manuscript form part of a common dataset for a programme of work dedicated to the ongoing translation, adaptation, and validation of the PSAS. Applications for use of the data can be made to the PSAS Chief Investigator (Dr. V. Fallon) on reasonable request. All applications will be ratified by the PSAS Working Group, and any publications resulting from analyses will have to credit the PSAS Working Group and the common dataset.
